# Effects of depot medroxyprogesterone acetate, the copper IUD and the levonorgestrel implant on testosterone, sex hormone binding globulin and free testosterone levels: ancillary study of the ECHO randomized clinical trial

**DOI:** 10.1186/s12905-024-02990-8

**Published:** 2024-03-08

**Authors:** G. Justus Hofmeyr, Mandisa Singata-Madliki, Joanne Batting, Yusentha Balakrishna, Chelsea Morroni

**Affiliations:** 1 Department of Obstetrics and Gynaecology, University of Botswana, Notwane Rd, Gaborone, Botswana; 2 Effective Care Research Unit, University of the Witwatersrand, Johannesburg, South Africa; 3 Effective Care Research Unit, Walter Sisulu University, East London, South Africa; 4 Effective Care Research Unit, Eastern Cape Department of Health, Bisho, South Africa; 5 Effective Care Research Unit, University of Fort Hare, Church St, East London, South Africa; 6 Biostatistics Research Unit, South African Medical Research Council, Durban, South Africa; 7 Botswana Harvard AIDS Institute Partnership, Gaborone, Botswana; 8 MRC Centre for Reproductive Health, University of Edinburgh, Edinburgh, UK

**Keywords:** Contraception, Depot medroxyprogesterone acetate, Copper intrauterine device, Levonorgestrel implant, Testosterone, Sex hormone binding globulin, Free testosterone, Randomized trial

## Abstract

**Background:**

Robust information on relative effects of hormonal contraceptives on endogenous androgens is important for understanding beneficial and adverse effects, method choice and development of new methods.

**Methods:**

In this ancillary study at the East London, South Africa site of the ECHO multicentre randomized trial, we compared effects of three contraceptive methods on serum androgen levels among contraceptive users aged 18 to 35 years. Participants were allocated by centrally-managed randomization to open label depot medroxyprogesterone acetate (DMPA-IM), copper intrauterine device (IUD) or levonorgestrel implant.

The primary outcome was free testosterone at 6 months.

**Results:**

We analysed stored baseline and 6-month serum samples in 398/615 participants (DMPA-IM 131/205, IUD 135/205 and implant 132/205). Median testosterone levels at baseline were DMPA-IM 0.82, IUD 0.9 and implant 0.87 nmol/L; at 6 months, DMPA 0.68 (lower than IUD, mean percentage difference 28.35, (*p* <  0.001), IUD 0.86 (unchanged) and implant 0.66, lower than IUD, mean percentage difference − 22.98, *p* <  0.001).

Median SHBG levels at baseline were DMPA 52.4, IUD 50.5 and implant 55.75 nmol/L; at 6 months, DMPA 40.65, lower than IUD (mean percentage difference 21.19, *p* = 0.005), IUD 49.1 (unchanged), and implant 23.35 nmol/L, lower than IUD (mean percentage difference − 50.04, *p* <  0.001 and than DMPA (mean percentage difference − 39.45, *p* <  0.001).

Free testosterone levels at baseline were DMPA 10, IUD 12 and implant 11 pmol/L; at 6 months, DMPA 11, less than IUD (mean percentage difference 13.53, *p* = 0.047), IUD 12 and implant 14, higher than IUD (mean percentage difference 14.15, *p* = 0.038) and than DMPA, (mean percentage difference 29.60, *p* <  0.001).

**Conclusions:**

This is the first randomized trial to show lower SHBG and higher free testosterone with the levonorgestrel implant than with DMPA, and contrasts with reports of increased SHBG with combined oral ethinyl estradiol/levonorgestrel use, and reduced androgens (and impaired sexual function) reported with the etonorgestrel implant. The higher free testosterone with the LNG implant might improve sexual function, mood and bone health as well as increasing side-effects such as acne and hirsutism, and is consistent with the greater sexual activity (with respect to multiple sex partners, new sex partner and unprotected sex) with the implant compared with DMPA documented in the ECHO study.

**ECHO trial registration:**

ClinicalTrials.gov, number NCT02550067 15/09/2015.

**Plain English summary:**

Contraception, or family planning, is central to the role of women in societies. It is most important to have accurate information on the relative side-effects of various contraceptive options in order to empower women to make informed choices regarding their preferred method.

Hormonal contraceptives contain various forms of the female sex hormones, estrogens and/or progestogens. These hormones have direct effects on the users, as well as modifying the levels of the users’ own circulating sex hormones, both the ‘female’ and the ‘male’ sex hormones (androgens).

In this study, consenting participants requesting contraception, were allocated randomly to receive either depot medroxyprogesterone acetate (DMPA-IM) a 3-monthly progestogen injection, the copper intrauterine device (IUD), a non-hormonal contraceptive inserted within the womb, or the levonorgestrel implant, a device placed under the skin which releases a progestogen for 5 years.

We measured the participants’ androgen levels after 6 months, and found for the first time that the active form of testosterone (free testosterone) was 29% higher with the implant than with DMPA-IM. The level with the IUD was intermediate, and significantly different from the other two methods.

This finding is relevant to the effects experienced by users of these methods, because free testosterone has effects on sexual function, bone health and mood, as well as on conditions such as acne and hair distribution patterns.

## Tweetable abstract

Higher free testosterone with LNG implant than DMPA intramuscular contraception may impact acceptance and sexual behaviour.

## Background

Hormonal contraception has complex effects on users’ endocrine systems. Apart from direct pharmacological effects of the exogenous contraceptive hormones, endogenous sex steroid hormone levels are altered. It is important to have accurate data on such effects, and in particular differential effects of alternative contraceptive methods, in order to be able to counsel users on the relative benefits and risks of alternative methods, to understand the potential clinical impact of these effects, and to guide future developments of contraceptive methods.

Previous consideration of androgenic effects of contraceptives such as oily skin, acne [1], hirsutism, android obesity, androgenic alopecia, unfavourable lipid profiles, diabetes and hypertension, has tended to focus on the direct androgenic effects of the exogenous progestins [2] rather than secondary effects on endogenous androgens. Minimizing androgenic side-effects is considered a key factor in the acceptability and continuation of hormonal contraception [3].

In addition to multiple physiological effects, sex steroid hormones have important neuropsychological and behavioural effects which are complex and poorly understood. Oral contraceptive discontinuation for depression or loss of libido appears to be less common with lower dose than the older high-dose formulations [4]. Low androgen levels have been associated with increased pelvic pain, dysmenorrhoea and headache [5]. Androgens are thought to increase libido, though results of previous studies are conflicting. Sexual function may be impaired by certain oral contraceptives and restored by androgen replacement [6]. Estrogens stimulate hepatic sex hormone binding globulin (SHBG) production [7]. Oral contraception with ethinyl estradiol combined with either levonorgestrel (LNG) or drospirenone increased SHBG and reduced testosterone and free testosterone levels. Addition of dehydroepiandrosterone (50 mg/day orally) normalized free testosterone [8]. Placebo-controlled randomized trials of testosterone treatment have shown improvement in hypoactive sexual desire disorder [7]. Testosterone has also been associated with sense of control and dominance.

Hormone-related effects on sexual behaviour and changes in sexual exposure during menstruation may affect susceptibility to sexually transmitted infections [9, 10].

In a previous randomized trial, we found reduced sexual activity among participants randomized to injectable progestogens versus the copper intrauterine device (IUD) [11, 12]. In the ECHO trial, reduced sexual activity and reduced condomless sexual activity was reported by participants allocated to depot medroxyprogesterone acetate intramuscular (DMPA-IM) compared with both the levonorgestrel implant and the IUD [13].

Previous research has found that some progestogenic contraceptives suppress testosterone levels. Subcutaneous DMPA 104 mg 12-weekly was associated with decreased total testosterone and sex hormone binding globulin (SHBG) to 26 weeks [14]. Lower levels of free testosterone were found among combined oral contraceptive than DMPA users [15]. Etonogestrel implants were associated with impaired sexual function, ascribed to suppressed androgen and estrogen levels [16].

Observational studies are intrinsically subject to confounding. The ECHO study presents a unique opportunity to compare testosterone and sex hormone binding globulin (SHBG) levels between participants allocated to DMPA-IM, the copper IUD and the levonorgestrel (LNG) implant in the context of a rigorous randomized clinical trial.

## Objectives

We compared testosterone, SHBG and free testosterone levels between women randomly allocated DMPA IM, the copper IUD or the LNG implant in order identify differences which might be associated with clinically important side effect profiles.

## Methods

This is an ancillary study of the ECHO study, limited to participants enrolled at the Effective Care Research Unit site in East London, South Africa between 1 March 2016 and 14 August 2017. The ECHO study protocol [17] and primary paper [13] have been published previously. Briefly, HIV uninfected participants requesting contraception aged 18 to 35 years who indicated that they had not used injectable hormonal contraception in the preceding 6 months, were allocated in parallel, in balanced blocks of 15–30, stratified by site, using an online randomization service, in 1:1:1 ratio to receive DMPA-IM 3-monthly, the copper T 380A IUD or the LNG implant, and were followed after 1 month then 3-monthly for 12 to 18 months. All participants gave written informed consent to participate. Participants were enrolled by research staff, and there was no blinding. Blood samples were collected at baseline and at the 3-monthly visits, separated on site and the serum stored at − 80 °C in the BARC-SA Bio Repository in Johannesburg. We chose the 6-month interval to allow adequate time for stabilisation of the effects of the hormonal contraceptives. For this ancillary study, a convenience sample size of all available specimens was used.

### Laboratory analysis

Laboratory tests were conducted by BARC-SA (Pty) Ltd. Testosterone and SHBG were measured at baseline and closest available sample to 6 months by chemiluminescent microparticle one-step immunoassay for quantitative measurement (CMIA) using the Abbott Architect analyser (sensitivity 0.05 and 0.02 nmol/L respectively; testosterone potential interference < 10%; SHBG: no detectable cross-reactivity). Free testosterone was calculated according to the method of Vermeulen et al. [18] The few results above or below the detection level of the assay were assigned the proxy value of the upper or lower limit of the assay respectively.

### Data analysis

The laboratory data were entered into an Excel spreadsheet, cleaned and merged with data from the original ECHO study database for analysis. Baseline data were compared to ensure that the comparability of the groups was not compromised by loss to follow-up, which was similar between groups. Core outcomes were not relevant to this hormonal study.

Baseline categorical demographic variables were compared between arms using Pearson’s chi-square or Fisher’s exact test, where applicable. Baseline continuous demographic variables were compared between arms using ANOVA. Normality was tested using the Shapiro-Wilk test. Since data was non-normally distributed, medians (with interquartile ranges) were reported for baseline, six-month and change from baseline values. Differences between baseline and six-month values for each allocated method were tested using the Wilcoxon signed-rank test to account for the paired data. To facilitate pairwise comparisons between the allocated methods, the data was log-transformed (natural log) and a mixed-effects linear regression model was fitted for each of the outcomes (testosterone, SHBG, free testosterone). The allocated method, time point and the interaction between allocated method and time point were included as fixed effects. The participant was included as a random effect to account for the within-subject correlation between repeated measures. The distribution of the residuals was visually assessed using quantile-quantile plots. The mean differences between allocated arms are presented as percentages with 95% confidence intervals (CIs). Results were considered significant for *p* <  0.05. It was not considered useful to adjust for daily variation in hormone levels [19] because these would be balanced out by the large sample size in each group.

## Patient and public involvement

Extensive patient and public involvement is documented in the primary ECHO study paper [13]. Community stakeholders were actively involved in the protocol design. Community advisory groups at each site provided input into conduct of the trial on an ongoing basis.

### Results

**Fig. 1:**
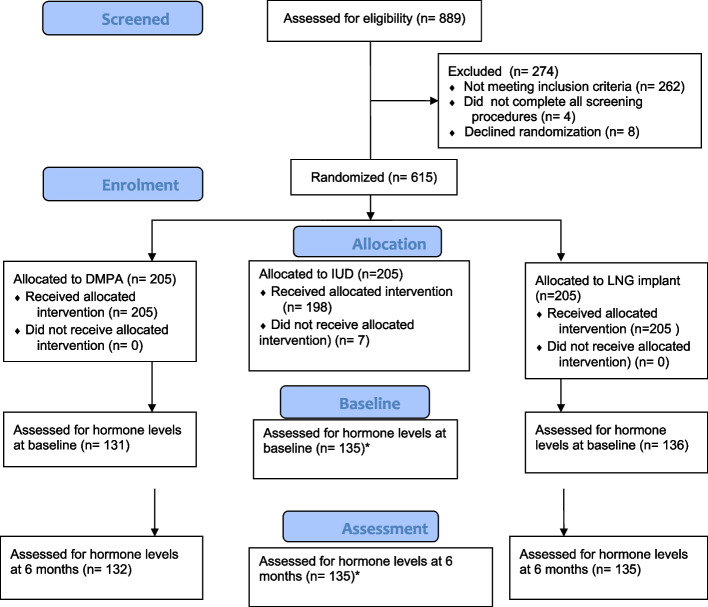
CONSORT flow diagram. *n = 2 did not receive the allocated intervention

Of 615 participants enrolled at the site, results were available for 398 (65%) participants both at baseline and at 6 months (Fig. 1).

Baseline Demographic data were similar between groups (Table 1).

**Table 1:** Baseline variables, expressed as mean values (standard deviation) or number, %

	DMPA			IUD			Implant			*p*
	n			n			n			
Age (years)	121	**25.0**	(4.3)	124	**25.4**	(4.6)	120	**24.5**	(4.7)	0.30
Never married	131	**122**	93.1%	135	**131**	97.0%	136	**132**	97.1%	0.23
Secondary school incomplete	131	**75**	57.3%	135	**63**	46.7%	136	**76**	55.9%	0.17
Earns income	131	**16**	12.2%	135	**22**	16.3%	136	**19**	14.0%	0.63
Nulliparous	131	**33**	25.2%	135	**40**	29.6%	136	**41**	30.2%	0.62
Regular menses	131	**76**	58.0%	135	**89**	65.0%	136	**80**	58.8%	0.34
Breastfeeding	131	**19**	14.5%	135	**12**	8.9%	136	**10**	7.4%	0.35
No Prior contraception	131	**3**	2.3%	135	**5**	3.7%	136	**6**	4.4	0.70
Fetal loss < 20 Weeks gestation	98	**16**	16.3%	95	**23**	24.2%	95	**22**	23.2%	0.35
Stillbirth	98	**2**	2.0%	95	**9**	9.5%	95	**4**	4.2%	0.07
Plan future children	131	**85**	64.9%	135	**96**	71.1%	136	**106**	77.9%	0.06
Age lastborn child	92	**3.1**	(2.9)	87	**3.6**	(3.1)	89	**3.0**	(2.4)	0.27
No alcohol	131	**71**	54.2%	135	**78**	57.8%	135	**66**	48.9%	0.34
Non smoker	131	**119**	90.8%	135	**116**	85.9%	135	**117**	86.7%	0.42
Has mobile phone	131	**129**	98.5%	135	**130**	96.3%	135	**131**	97.0%	0.64

In the main trial, random testing of baseline blood of 157/615 participants for evidence of recent hormonal contraceptive exposure found quantifiable medroxyprogesterone acetate in 85 (54%), levonorgesterel in 9 (5.7%), norethisterone in 7 (4.5%) and etonogestrel in 1 (0.6%). Given the potential for distortion of baseline hormone levels due to prior contraceptive exposure, we have primarily compared absolute differences between randomized groups at 6 months as well as change from baseline. Prior exposure to progestogen contraception would be expected to be similar between randomly allocated groups, and would result in under-estimation of the changes observed in the progestogen groups. Hormonal results are shown in Tables 2 and 3.

**Table 2:** Hormonal results expressed as median (interquartile range, IQR) values

Treatment	n	Baseline median (IQR)	n	6 months median (IQR)	n	Median change (6 m vs baseline, IQR)	*p*-value^a^
Testosterone, nmol/L							
DMPA	131	0.82 (0.56, 1.2)	132	0.68 (0.5, 0.88)	131	−0.16 (− 0.35, 0.04)	< 0.001
IUD	135	0.9 (0.66, 1.18)	135	0.86 (0.68, 1.11)	135	0.01 (−0.19, 0.14)	0.337
Implant	135	0.87 (0.64, 1.26)	135	0.66 (0.51, 0.85)	133	−0.23 (−0.48, − 0.03)	< 0.001
Sex Hormone Binding Globulin, nmol/L							
DMPA	131	52.4 (38.4, 74.1)	132	40.65 (31.55–53.9)	131	−9.6 (−21.8, 0.4)	< 0.001
IUD	135	50.5 (34.7, 76)	135	49.1 (36.2–71.1)	135	3 (−13, 15.5)	0.618
Implant	132	55.75 (34.45, 81.9)	134	23.35 (18.1–33.6)	132	−27.2 (−45.85, −12.05)	< 0.001
Free testosterone, pmol/L							
DMPA	131	10 (7, 16)	131	11 (8, 15)	131	0 (−3, 3)	0.516
IUD	135	12 (9, 16)	135	12 (9, 17)	135	0 (−4, 2)	0.225
Implant	136	11 (8, 16)	132	14 (10, 18.5)	132	3 (−1.5, 6)	<0.001

^a^ p-value from Wilcoxon signed-rank test for difference between baseline and six-month values. *DMPA*, Depot medroxyprogesterone acetate, *IUD* Copper intrauterine device, Implant – levonorgestrel implant

**Table 3:** Pairwise comparisons of hormonal results between allocated methods expressed as mean percentage differences (95% confidence interval (CI))

Treatment	Baseline mean % difference (CI)	p-value	6 months mean % difference (CI)	p-value	Change mean % difference (CI)	p-value
Testosterone						
IUD vs DMPA	7.32 (−3.7, 19.6)	0.202	28.35 (15.19, 43.02)	< 0.001	19.60 (7.24, 33.39)	0.001
Implant vs DMPA	10.09 (−1.21, 22.68)	0.082	−1.14 (− 11.27, 10.15)	0.836	-10.20 (-19.49, 0.18)	0.054
Implant vs IUD	2.58 (−7.88, 14.22)	0.642	−22.98 (−14.23, −30.83)	< 0.001	−24.91 (-32.63, -16.31)	<0.001
Sex Hormone Binding Globulin						
IUD vs DMPA	−7.73 (−19.23, 5.41)	0.236	21.19 (6.10, 38.42)	0.005	31.34 (15.84, 48.91)	<0.001
Implant vs DMPA	0.37 (−12.19, 14.73)	0.957	−39.45 (−30.82, − 47)	< 0.001	−39.67 (− 46.82, − 31.56)	< 0.001
Implant vs IUD	8.78 (− 4.75, 24.23)	0.214	−50.04 (− 42.96, − 56.23)	< 0.001	−54.07 (− 59.48, − 47.94)	< 0.001
Free testosterone						
IUD vs DMPA	14.91 (1.37, 30.26)	0.030	13.53 (0.15, 28.7)	0.047	-1.20 (-11.87, 10.77)	0.836
Implant vs DMPA	7.70 (−4.97, 22.06)	0.245	29.60 (14.28, 46.97)	< 0.001	20.34 (7.29, 34.97)	0.002
Implant vs IUD	−6.28 (−17.22, 6.12)	0.306	14.15 (0.76, 29.33)	0.038	21.80 (8.68, 36.50)	0.001

### Testosterone

Baseline median (interquartile range) testosterone levels were similar between the three groups (DMPA 0.82 (0.56, 1.2), IUD 0.9 (0.66, 1.18), implant 0.87 (0.64, 1.26) nmol/L). At 6 months, median testosterone levels were significantly reduced for DMPA (0.68 (0.5, 0.88)) and the levonorgestrel implant (0.66 (0.51, 0.85) nmol/L), which were similar, and both were significantly lower (mean percentage differences 28.35 and − 22.98, *p* < 0.001 and *p* = 0.001 respectively) than that for the IUD (0.86 (0.68, 1.11) nmol/L) which was not significantly changed from baseline.

### Sex hormone binding globulin (SHBG)

Baseline median SHBG levels were similar between the three groups (DMPA 52.4 (38.4, 74.1), IUD 50.5 (34.7, 76) and implant 55.75 (34.45, 81.9) nmol/L). At 6 months, median SHBG levels in the IUD group were unchanged (49.1 (36.2–71.1) nmol/L). Levels for DMPA and the implant were significantly reduced from baseline. That for DMPA (40.65 (31.55–53.9) nmol/L) was significantly lower than the IUD (mean percentage difference 21.19, *p* = 0.005). That for the implant (23.35 (18.1–33.6) nmol/L) was significantly lower than both the IUD (mean percentage difference − 50.04, *p* < 0.001) and DMPA (mean percentage difference − 39.45, p < 0.001).

### Free testosterone

Baseline median free testosterone levels were similar between the three groups (DMPA 10 (7, 16), IUD 12 (9, 16) and implant 11 (8, 16) pmol/L). At 6 months, median free testosterone levels for DMPA (11 (8, 15)) was lower (mean percentage difference 13.53, *p* = 0.047) and the implant (14 (10, 18.5) higher (mean percentage difference 14.15, *p* = 0.038) than the IUD (12 (9, 17). The value for the implant was higher than for DMPA (mean percentage difference 29.60, *p* < 0.001).

## Discussion

This is to our knowledge the first randomized trial to quantify the differences in levels of testosterone, SHBG and free testosterone in young participants randomly allocated to receive DMPA, the LNG implant, or non-hormonal contraception (the IUD). Total testosterone levels of those allocated to DMPA and to LNG implant were significantly lower than for the IUD after 6 months. The striking finding was the significantly greater reduction in SHBG with the implant than with DMPA, which resulted in the free testosterone being 29% higher with the implant than with DMPA. This large difference may have meaningful consequences in terms of side-effects such as acne and hirsutism [20], as well as beneficial effects on sexual function, mood and bone health. The possible association of free testosterone with libido is consistent with the finding in the main Echo Trial [13] of significantly higher reports in the LNG implant than the DMPA group for multiple sex partners, new sex partner, unprotected sex and no condom used for the last sex act. The differences in reported unprotected sex are also consistent with an ancillary study at three of the ECHO sites which found prostate-specific antigen levels in cervical samples to be more frequent in women allocated to the levonorgestrel implant and the Cu IUD than to DMPA [21]. The markedly reduced SHBG with the levonorgestrel implant is in sharp contrast to levonorgestrel containing oral contraception which is associated with increased SHBG [22], probably due to estrogenic stimulation of hepatic SHBG production. The etonogestrel implant has been associated with impaired sexual function ascribed to suppressed estrogen and androgen levels [16]. In contrast, increased free testosterone with the levonorgestrel implant might differentiate user acceptability between the two implant formulations.

Given the findings of this study, it is important not to assume similar side-effect profiles of progestogen contraceptives, but to keep in mind the potential for differential effects of the progestogens on endogenous hormone levels.

The results should be generalisable to similar, low-risk populations.

### Limitations

As sampling was at the time of the DMPA administration visit, the DMPA results would reflect the nadir DMPA effect, and the implant results would represent the steady state effect at 6 months. This study could not assess possible post-injection peak effects of DMPA, and thus may have under-estimated differences between the methods.

Spot checks conducted during the main study indicated that a proportion of women had evidence of persistent MPA or norethisterone levels or recent oral contraceptive pill use. Considerable discrepancies between self-reported and biologically confirmed prior contraceptive exposure have been reported in other studies [23]. Use of oral contraception was permitted up to the day preceding enrolment. It is likely that some women in all groups had some testosterone and SHBG suppression at baseline. These effects are likely to be similar between groups due to the stringent randomized methodology. The measured reductions in testosterone and SHBG in the hormonal methods groups may be an under-estimate of the true suppressive effect of the methods. The effects of hormonal contraception may be affected by clinical characteristics such as body mass index and medication. Because the objective of the study was to measure comparative differences between contraceptive methods rather than absolute levels, and because stringent randomization and large sample size would balance such clinical characteristics between groups, as confirmed by the similar baseline characteristics, it was not necessary to adjust for confounding clinical characteristics.

## Conclusions

We have compared testosterone, SHBG and free testosterone levels between participants allocated to three contraceptive methods in the context of a robust randomized trial. The significantly higher levels of free testosterone with the LNG implant than with DMPA-IM may have important clinical implications in terms of differential physiological, psychological and behavioural effects, side-effects and acceptability of these methods, and the tailoring of method choice according to user clinical profiles.

## Data Availability

The data that support the findings of this ancillary study of the ECHO Study are available from the corresponding author upon reasonable request. Access will be granted if the concept is evaluated to have scientific merit and if sufficient data protections are in place. As of the time of publication, data access applications are in process with the governing institutional review boards of the ECHO Study to make de-identified data from the primary ECHO dataset publicly available.

## Abbreviations

ECHO: Evidence for contraceptive options and HIV Outcomes
DMPA-IM: Depot medroxyprogesterone acetate intramuscular
IUD: Intrauterine device
LNG: Levonorgestrel
